# Quantitative description of a contractile macromolecular machine

**DOI:** 10.1126/sciadv.abf9601

**Published:** 2021-06-11

**Authors:** Alec Fraser, Nikolai S. Prokhorov, Fang Jiao, B. Montgomery Pettitt, Simon Scheuring, Petr G. Leiman

**Affiliations:** 1 Department of Biochemistry and Molecular Biology, Sealy Center for Structural Biology and Molecular Biophysics (SCSB), The University of Texas Medical Branch at Galveston, Galveston, TX 77555, USA.; 2 Department of Anesthesiology, Weill Cornell Medicine, 1300 York Avenue, New York, NY 10065, USA.; 3 Department of Physiology and Biophysics, Weill Cornell Medicine, 1300 York Avenue, New York, NY 10065, USA.

## Abstract

Contractile injection systems (CISs) [type VI secretion system (T6SS), phage tails, and tailocins] use a contractile sheath-rigid tube machinery to breach cell walls and lipid membranes. The structures of the pre- and postcontraction states of several CISs are known, but the mechanism of contraction remains poorly understood. Combining structural information of the end states of the 12-megadalton R-type pyocin sheath-tube complex with thermodynamic and force spectroscopy analyses and an original modeling procedure, we describe the mechanism of pyocin contraction. We show that this nanomachine has an activation energy of 160 kilocalories/mole (kcal/mol), and it releases 2160 kcal/mol of heat and develops a force greater than 500 piconewtons. Our combined approach provides a quantitative and experimental description of the membrane penetration process by a CIS.

## Teaser

Contractile injection systems generate 500 pN forces for membrane puncture by a domino-like collapse of sheath subunits.

## INTRODUCTION

**Fig. 1:**
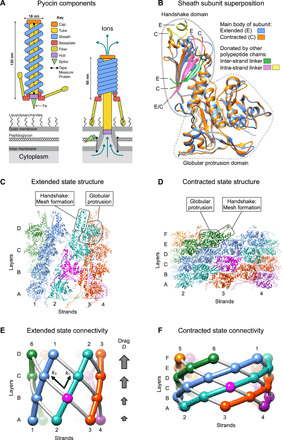
Structure of the end states and parametrization of the contraction reaction. (**A**) Schematic showing the main components and size of the pyocin particle free in solution (extended sheath) and attached to the cell surface (contracted sheath). (**B**) Superposition of sheath subunits in the extended and contracted states. The β sheet of the handshake domain is completed by the inter- and intra-strand linkers that belong to adjacent polypeptide chains. The main body of the subunit in the extended and contracted conformations is colored in dodger blue and orange, respectively. The fragments originating from the neighboring polypeptide chains are shown in distinct colors and labeled with the letters E and C, which correspond to the extended and contracted conformations of the sheath, respectively. (**C**) Structure of a four-layer fragment of the pyocin sheath complex in the extended state (*5*). Each strand has a distinct color. One subunit is colored magenta to serve as a reference point. The strands are numbered, and the layers are labeled with letters. The boxed handshake domain lacks β sheet components from adjacent subunits for clarity. (**D**) Structure of a six-layer fragment of the pyocin sheath in the contracted state (*5*). The color code and labeling nomenclature are as in (C). The boxed handshake domain lacks β sheet components from adjacent subunits for clarity. (**E** and **F**) Diagrams demonstrating the topology and connectivity of polypeptide chains comprising the sheath in the extended and contracted states. The spheres represent subunit COMs. The sheath strands (intra-strand connections) are shown with colored tubes. The gray tubes indicate inter-strand connections. Also shown are the intra- and inter-strand coupling constants *k*_1_ and *k*_2_ and the drag parameter *D*.

Contractile injection systems (CISs), which include the bacterial type VI secretion system (T6SS), bacteriophage tails, R-type pyocins, and other tailocins, function to penetrate bacterial and eukaryotic membranes (*1*–*4*). The universally conserved part of CISs consists of an external contractile sheath, an internal rigid tube, and a baseplate (Fig. 1A). The tube and the sheath are made up of sixfold symmetric layers of subunits stacked upon each other with a twist, forming a helical structure. The initial extended conformation of the complex represents a high energy metastable state (*5*, *6*). The sheath contracts to about half of its original length upon activation through a specific stimulus originating at the baseplate, e.g., attachment to the target cell surface, or spontaneously when subjected to stress or upon long storage (*7*, *8*). The baseplate-distal end of the tube and the sheath are fixed to each other with a capping protein (Fig. 1A). Consequently, contraction of the sheath results in the motion of the tube toward and through the target cell membrane (Fig. 1A) (*7*). This process is aided by a spike-shaped protein located at the baseplate-proximal end of the tube (Fig. 1A) (*9*, *10*). The membrane-attacking tip of the spike protein is stabilized by an iron or zinc atom (*9*, *10*).

The atomic structure of the sheath-tube complex of the R-type pyocin, T6SS, the Photorhabdus Virulence Cassette, and its closely related Serratia Antifeeding Prophage in the extended and contracted states has been determined by cryo–electron microscopy (cryo-EM) (*5*, *11*–*14*). Of these, the pyocin has the simplest architecture. Its sheath subunit consists of two domains, one of which is a component of an interconnected fishnet-like mesh that envelopes the tube [termed the “handshake domain” (*11*)], whereas the other forms a globular protrusion positioned at each node of this mesh (Fig. 1, B to F). Other CISs build on top of this architecture by adding one or two domains to the protrusion domain and retaining all the other features of the pyocin sheath (*15*). In all these systems, the structure of the individual subunit and the topology of the mesh connecting the handshake domains are preserved in both the contracted and extended states of the sheath (*5*, *11*–*14*). The structure of the tube is very similar in all CISs (*5*, *11*–*14*).

Despite the knowledge of the atomic structures of several CISs in the two end states, the mechanism by which chemical energy stored in the extended state is converted into the motion of the tube remains poorly understood. Previous theoretical work, which was performed before atomic structures of the sheath-tube complex became available, provides excellent insight into the contraction process but lacks quantitative details (*6*, *16*–*18*). The available experimental data are sparse and somewhat contradictory. The enthalpy of sheath contraction measured for T4 ghosts (phage particles lacking DNA in which the sheath comprises less than 10% of the total material) varied by a factor of 2 depending on whether contraction was triggered by heat or urea (*19*). The activation energy of urea-induced contraction was found to be negative. The upper boundary for the time scale of contraction comes from studies of green fluorescent protein–labeled T6SS sheaths that are long enough (~10 times longer than phage tails) to be visualized in a fluorescence microscope (*20*). The actual time scale is nevertheless unknown as contraction occurred faster than the 5-ms framerate of the microscope camera (*20*).

A few contraction intermediates of T4 and other phages have been captured in the electron microscope over the years, showing that the in vitro and in vivo triggered contractions start at the baseplate and propagate through the length of the sheath as a wave (*8*, *16*, *21*, *22*). The sheath forms a narrow Christmas tree–like structure in phages targeting Gram-negative bacteria (*16*) but contains a sharper transition from a wider contracted part to a narrower extended region in phages targeting Gram-positive hosts (*8*, *21*, *22*). In the latter case, these intermediates are long-lived and could represent a functional state associated with the enzymatic digestion of the cell wall by enzymes located at the tip of the tail tube, an event that must precede the completion of sheath contraction during infection of a Gram-positive bacterium (*8*).

Recently, a computational approach that modeled the T4 sheath using Kirchhoff’s rod theory with parameters derived from molecular dynamics (MD) simulations of a short fragment of the sheath has been presented (*23*). The elastic body calculations were parametrized to match the enthalpy of T4 sheath contraction (*19*) and the contraction time scale of the T6SS (*20*). Contraction was predicted to proceed via a rapid rotation of sheath subunits before their translation (*23*, *24*). Such a sequence of events is incompatible with maintaining the integrity of the sheath subunit, which was implied but not validated in the approach. Furthermore, the model predicts that the free energy profile of the contraction process has an exponential form and a zero activation energy. Consequently, the forces developed in the second half of the contraction process when the spike-tube complex comes into contact with the host membrane are vanishingly small and thus are insufficient for membrane puncture.

Here, we present a new modeling procedure that describes the free energy profile for the contraction process of the R-type pyocin sheath-tube complex in atomic detail. We developed a set of solution biophysics and single-molecule experiments that characterize the activation energy *E*_a_, the enthalpy of contraction, and the effective spring constant for the contracted sheath. Our modeling procedure correctly predicts, and solution biophysics measurements confirm the properties of pyocin mutants with an altered transition state structure.

## RESULTS

### Sheath contraction requires both theoretical and experimental characterization

Sheath contraction is a physicochemical reaction in which a change in the chemistry of the reactants (interactions between sheath subunits) is converted into mechanical work (motion of the tube). We characterize this process by a combination of atomic structure–based modeling and experimental measurements. Modeling aims to generate realistic sets of atomic structures that describe the contraction process. From this, the model predicts the following experimentally measurable parameters: the total free energy change, the activation energy, and the forces generated throughout the contraction. Furthermore, the model describes the structure of the highest energy state (the transition state), which enables the manipulation of the activation energy by targeted mutagenesis. Therefore, modeling is integral in guiding our experiments, and consequently, the model must be described first, with experiment to follow.

### Parametrization of the contraction reaction

In finding the most probable contraction pathway, a set of intermediate structures that describe the transition between the extended and contracted states, we must take into account both the structural constraints and the energetics of the system. Considering that the mesh-like connectivity of the handshake domains (Fig. 1, C to F) and the overall structure of the sheath subunit (Fig. 1B) are preserved in both the extended and contracted states, as well as the fact that the mesh linkers are integral parts of the handshake domain (Fig. 1B), we assumed that the fold of the subunit and, consequently, the connectivity of subunits are maintained throughout the contraction event. Furthermore, the architecture of the sheath’s mesh and the near-perfect roundness of the sixfold symmetric tube, whose structure does not change during contraction, do not allow the quaternary structure of the sheath-tube complex to substantially deviate from the sixfold symmetry. Hence, we further assumed that contraction occurs in a sixfold symmetric manner.

**Fig. 2:**
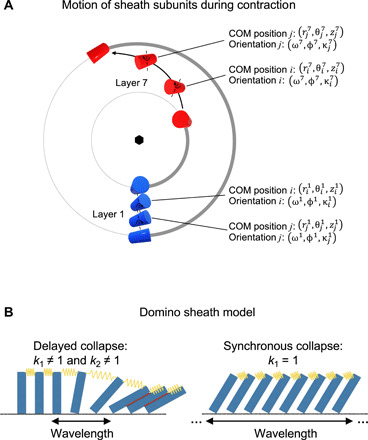
The geometry and macroscopic approximation of sheath contraction. (**A**) A top-view projection of the motion of the baseplate-proximal (layer 1, blue) and a middle (layer 7, red) sheath subunit belonging to the same strand (thick gray line) during contraction. The subunits are shown schematically as cylinders. The radial motion is exaggerated for clarity. (**B**) A row of dominoes connected by springs is a one-dimensional representation of the sheath. The red lines indicate favorable interactions between dominoes (or sheath subunits) that are realized in the lowest energy state (contracted state). The value of the spring constant defines whether the collapse of these dominos (or sheath contraction) is synchronous or delayed. In a delayed collapse, the contraction wavelength spans a finite number of subunits. In a synchronous collapse, the contraction wavelength is infinite.

Given these constraints, the instantaneous position of any sheath subunit in a contraction intermediate can be described by a set of six parameters (*r*, θ, *z*, ω, ϕ, κ) where (*r*, θ, *z*) are the cylindrical coordinates of the subunit’s center of mass (COM) and (ω, ϕ, κ) are the set of polar angles describing the rotation of the subunit relative to the extended state. Notably, for a given subunit, the rotation axis, which is defined by the angles (ω, ϕ), is fixed throughout contraction in the COM reference frame (Fig. 2A). Accordingly, the rotation of the subunit can be effectively described by a single angle κ that spans a range of values from 0 to κ_cnt_. Furthermore, given the constraint of preserving the connectivity and integrity of the handshake domains, the (*r*, θ, *z*, κ) parameters must be linearly related. Substantial deviations from this linearity cause the handshake domains to disintegrate. Accordingly, we express this linear relationship by the means of a “contracted fraction” parameter λ, with λ = 0 and λ = 1 corresponding to the extended and contracted states, respectively, such that ∀ (*r*, θ, *z*, κ) ∃ λ ∈ [0, 1] ∣ (*r*, θ, *z*, κ) = (1 − λ) × (*r*_ext_, θ_ext_, *z*_ext_, 0) + λ (*r*_cnt_, θ_cnt_, *z*_cnt_, κ_cnt_). Last, the contracted fraction of the entire sheath structure (our reaction coordinate) can be represented as an average of the contracted fractions of all sheath subunits.

An important consequence of the rigid body approximation for the motion of sheath subunits is that changes in the energetics of the sheath-tube complex are dominated by changes in interfacial interactions between subunits. The free energy of these interactions can be evaluated by summing the products of atomic solvent accessibilities and the free energy of solvation for every atom comprising the interface (*25*). In addition, hydrogen bonds, salt bridges, and disulfide bonds (not applicable here) contribute to the interfacial energetics. Such an algorithm is implemented in the Protein Interfaces, Surfaces and Assemblies (PISA) software package, which was designed for the identification of biologically meaningful interfaces in protein crystals (*26*). Here, we use PISA to calculate the total solvation energy of all contraction intermediates of the sheath-tube complex.

### The search for a contraction pathway

**Fig. 3:**
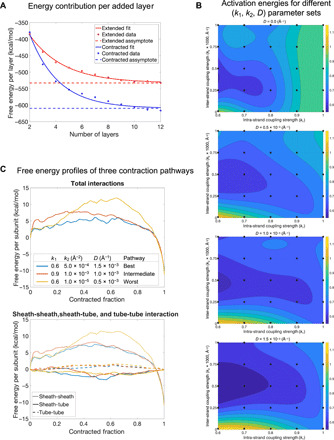
DMAD analysis of the 12-layer sheath-tube fragment. (**A**) Free energy contribution per layer of sheath subunits as a function of the number of layers in the extended and contracted states. (**B**) Activation energies (as a fraction of Δ*G*) for 100 contraction pathways are plotted on the (*k*_1_, *k*_2_) planes for four different values of the drag parameter *D*. A third-degree polynomial surface is fitted to the data points. (**C**) Free energy profiles of contraction pathways with the lowest, intermediate, and highest activation energies. Top: The total sum of all interactions. Bottom: The interactions of each of the three component pairs: sheath-sheath, sheath-tube, and tube-tube.

The most probable contraction pathway should exhibit the lowest activation energy and be devoid of substantial local minima since semi-contracted intermediates of pyocins have not been observed (*27*). We used a two-step procedure to identify such a pathway. First, we analyzed the contraction energetics of the smallest fragment of the sheath-tube complex in which the relative influence of the solvent-exposed, terminal segments can be neglected (a 12-layer, 144-subunit segment of the sheath-tube complex) (Fig. 3A). Then, we extrapolated the best contraction pathway to the full-length 28-layer structure, taking into account the structural constraints of the system.

Different contraction pathways were generated by varying three parameters that determine the physical properties of the sheath structure: two spring-like constants *k*_1_ and *k*_2_ that describe the transfer of momentum between sheath subunits mediated by intra- and inter-strand linkers, respectively, and a drag-like parameter *D* that retarded the motion of baseplate-distant subunits and accounted for frictional and viscosity forces in the system (Fig. 1, E and F). The range of values for each of the three parameters (*k*_1_, *k*_2_, *D*) was chosen such that the integrity of the handshake domains was maintained throughout contraction. Implementation of the above procedure as an algorithm constitutes the Domain Motion in Atomic Detail (DMAD) modeling method (see Materials and Methods and data S1).

One hundred contraction pathways, sampled as 5 × 5 matrices of (*k*_1_, *k*_2_) pairs for four different values of *D*, were generated (Fig. 3B and data S2). The pathways displayed different free energy profiles and activation energies (Fig. 3C). Notably, sheath-sheath subunit interactions dominated the energetics of the system while the initial sheath-tube subunit interfaces had negligibly small association energies (Fig. 3C). Optimal pathways occurred in a distinct region of the (*k*_1_, *k*_2_) plane for all values of the *D* parameter (Fig. 3B). This region is defined by low-to-intermediate values for both the intra- and inter-strand coupling. These contraction pathways can be described as “delayed” or “wave-like,” akin to the collapse of dominoes connected by weak springs and surrounded by a viscous medium (Fig. 2B). These pathways are characterized by a “contraction wavelength” defined as the smallest number of layers between near–fully contracted and near–fully extended subunits in an intermediate structure. The “synchronous” contraction pathway is realized when the intra-strand coupling constant *k*_1_ = 1, which corresponds to an infinitely stiff spring in the domino model (Fig. 2B). Its contraction wavelength is infinitely long, and its activation energy is higher than that of most delayed contraction pathways.

**Fig. 4:**
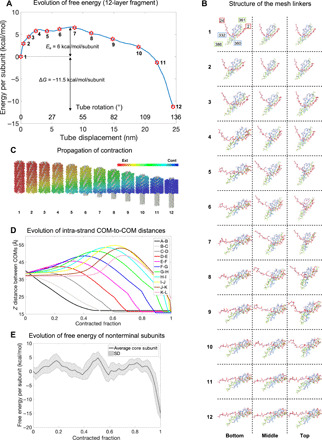
DMAD-derived contraction pathway of the 12-layer fragment. (**A**) Free energy profile of the best contraction pathway of the 12-layer fragment. (**B**) Evolution of the structure of the intra- and inter-strand linkers throughout the contraction process for the best pathway. The three columns illustrate the bottom (baseplate-proximal), middle, and top (baseplate-distal) layers of the structure. Each row shows one of the 12 conformations labeled with red stars in (A). Each polypeptide chain is in a distinct color. Residues 2 to 24 extend from the left-adjacent strand and one layer above subunit (inter-strand connectivity, red). Residues 361 to 386 extend from the subunit in the same strand and one layer above (intra-strand connectivity, green). (**C**) Propagation of contraction throughout the sheath. Sheath subunits are colored according to their contracted fraction with a color key given in the top right corner of the panel. The free energies of the 12 intermediates shown are indicated with red stars in (A). (**D**) Evolution of the vertical component of the distance between COMs of sheath subunits belonging to the same strand in the 12-layer fragment throughout the best contraction pathway. Layers are labeled with consecutive letters A to L starting from the baseplate (layer A). (**E**) Average free energy profiles of eight nonterminal subunits (belonging to layers 3 to 10 in the 12-layer fragment) are plotted as a function of their contracted fraction.

The best contraction pathway of the 12-layer fragment had an activation energy of 6 kcal/mol per sheath subunit (Fig. 4A and movies S1 and S2). All structures in the pathway were characterized by a reasonable-to-good geometry as verified by MolProbity (table S1) (*28*). The structure of the handshake domain was maintained throughout contraction (Fig. 4B). Notably, the contraction wavelength was longer than the length of the structure (Fig. 4C). During contraction, the distances between the COMs of the sheath subunits and, consequently, the length of the sheath strands increased before collapsing into the compact contracted state (Fig. 4D). The sheath strands thus became hyperextended, and the action of the sheath-tube system resembled that of a ballista.

### Contraction of the full-length structure

**Fig. 5:**
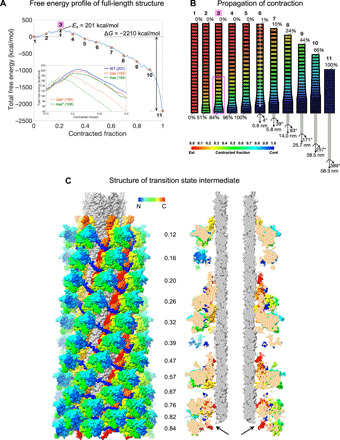
Free energy profile of contraction and the structure of the transition state. (**A**) DMAD-derived evolution of the free energy of the sheath-tube complex during contraction. Eleven red stars mark the free energy of conformations shown schematically in (B). Intermediate 3 (magenta background) is the transition state. The inset shows a fragment of free energy profiles for sheath mutants with inter-strand linkers carrying two and four additional amino acids that are labeled 2aa and 4aa, respectively. The solid lines correspond to the WT simulation and modifications of the inter-strand constant *k*_2_ described by the following (*k*_1_, *k*_2_, D) parameters: WT (0.7, 2.5 × 10^−4^ Å^−2^, 5 × 10^−4^ Å^−1^), 2aa (0.7, 1.4 × 10^−4^ Å^−2^, 5 × 10^−4^ Å^−1^), and 4aa (0.7, 8 × 10^−5^ Å^−2^, 5 × 10^−4^ Å^−1^). The dashed lines to modification of both *k*_1_ and *k*_2_: 2aa* (0.665, 2.375 × 10^−4^ Å^−2^, 5 × 10^−4^ Å^−1^) and 4aa* (0.63, 2.25 × 10^−4^ Å^−2^, 5 × 10^−4^ Å^−1^). All profiles shown in the inset have been smoothed for clarity. (**B**) Propagation of contraction throughout the pyocin structure. The sheath layers are colored according to their contracted fraction using the color code given in the bottom part of the panel. The width and height of the sheath layers are also adjusted to match the contracted fraction. The transition state intermediate 3 is labeled with a magenta background. The magenta rectangle highlights the 12 baseplate-proximal layers of the transition state shown in (C). The semitransparent white line (intermediate 6) represents the contraction wavelength. (**C**) Structure of the 12 baseplate-proximal layers of the full-length sheath-tube complex in the transition state. The color of each sheath subunit varies along the polypeptide chain as a continuous spectrum with the N terminus in blue and the C terminus in red. The tube is colored gray. The cutaway view panel on the right shows baseplate-proximal sheath subunits that have dissociated from the tube (black arrows), but the tube has yet to move. The contracted fraction of each sheath layer is given between the panels.

Simulations of the 12-layer sheath-tube fragment revealed that given a set of (*k*_1_, *k*_2_, *D*) parameters, the free energy of nonterminal sheath subunits (layers 3 to 10 in the 12-layer structure) is independent of the subunit’s position within the strand and is uniquely determined by its contracted fraction (Fig. 4E). Similarly, given a set of (*k*_1_, *k*_2_, *D*) parameters, the contribution of the four terminal layers (the two bottom layers and the two top layers), in which not all intersubunit interfaces are engaged in the contracted state, is the same in a structure of any length. These observations make it possible to extrapolate a contraction pathway of the 12-layer fragment to that of the full-length 28-layer structure and to obtain a free energy profile of contraction for the full-length sheath (see Materials and Methods). At the same time, pathways in which the vertical distance between neighboring subunit’s COMs exceeds 55 Å, the maximal value found for the 12-layer fragment (Fig. 4D), cannot be realized as this is incompatible with maintaining the structural integrity of the sheath. For this reason, all but the lowest-drag pathways are prohibited for the full-length pyocin structure. Furthermore, both the drag *D* and inter-strand constant *k*_2_ had to be rescaled to take into account the longer paths traveled by baseplate-distal sheath subunits in the full-length structure (see Materials and Methods). After these considerations, optimal parameters from the 12-layer fragment simulations were used, namely, *k*_1_ = 0.7 (dimensionless), *k*_2_ = 2.5 × 10^−4^ Å^−2^, and *D* = 5 × 10^−4^ Å^−1^. This contraction pathway had an activation energy of 201 ± 12 kcal/mol, which corresponded to ~9% of the total energy released (2210 kcal/mol) (Fig. 5A).

### Structure of the transition state and the wavelength of contraction

Sheath contraction is thought to be triggered by a large conformational change of the baseplate, which propagates through the sheath via direct and near–rigid body interactions between the baseplate and baseplate-proximal sheath subunits (*13*, *27*, *29*). The transformation of the baseplate likely provides the activation energy necessary to reach the transition state. Accordingly, a putative transition state should exhibit baseplate and baseplate-proximal sheath subunits in a near-contracted state. In addition, the structure of the transition state should allow for a return to its original, fully extended state.

In the transition state predicted by the DMAD methodology, the baseplate-proximal (“bottom”) sheath subunits of the sheath are ~84% contracted, whereas the baseplate-distal (“top”) subunits are fully extended (intermediate 3 in the pathway in Fig. 5, A and B). As a consequence, the bottom sheath subunits separate from the tube, whereas the top part of the sheath remains in the extended state and interacts with the tube (Fig. 5C). This intermediate is comparable to the structure of the phage T4 tail with its baseplate in the post-attachment state and the tail not yet contracted, which has been imaged attached to the cell surface by cryo–electron tomography (*30*). Further along the contraction pathway is an intermediate in which the fifth subunit from the bottom and the top subunit are ~98% and ~1% contracted, respectively (intermediate 6 in Fig. 5, A and B). This distance corresponds to the smallest number of layers between near–fully contracted and near–fully extended subunits in an intermediate structure. Thus, the contraction wavelength spans 24 layers and is approximately equal to the size of the entire complex (Fig. 5B).

### Probing contraction with solution biophysics

**Fig. 6:**
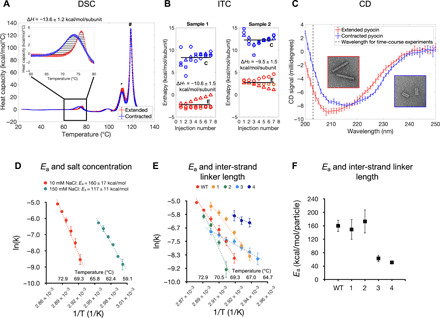
Characterization of contraction reaction using solution biophysics. (**A**) DSC profiles of extended and contracted pyocins. The inset highlights the temperature range where temperature-induced sheath contraction occurs. The enthalpy of contraction is the integrated area of the striated region. The asterisk (*) and hash (#) symbols indicate the peaks associated with heat absorbed during the denaturation of the tube and sheath, respectively. The average and SDs of three technical replicates are plotted. Error bars represent the SD. The experiment was repeated for two biological replicates. (**B**) Enthalpy of pH-induced pyocin contraction is measured using an inverted ITC setup. The extended (E) and contracted (C) pyocins were titrated into a cell containing a pH 2.5 buffer. Three replicates of both, extended and contracted samples for two biological replicates, were measured (each titration is labeled with Δ, ○, or □ symbols). The averages are shown with solid lines. (**C**) CD spectra of extended and contracted pyocins. The vertical dashed line indicates the wavelength used in time-course measurements. The insets show EM images of the samples used in these experiments. The curves are averages of three technical and three biological replicates. Error bars correspond to the SD. (**D**) Arrhenius plot of temperature-dependent contraction rates of the WT pyocin for two buffer conditions. Data points are averages of three technical and three biological replicates. Error bars represent the SD. (**E**) Arrhenius plot of temperature-dependent contraction rates for WT and four sheath mutants. Data points are averages of three technical and two biological (three for WT) replicates. Error bars represent the SD. (**F**) Activation energy plot as a function of the inter-strand linker length. Error bars represent 95% confidence interval for the Arrhenius fit.

**Fig. 7:**
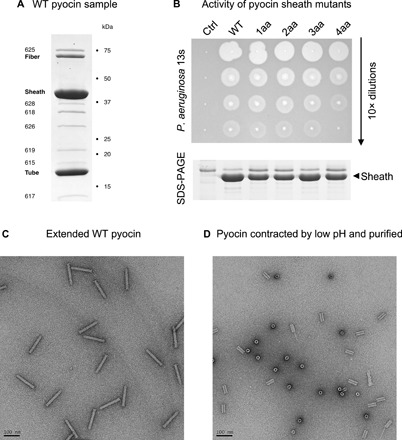
Biophysical and functional characterization of the WT pyocin and its sheath mutants. (**A**) Coomassie-stained SDS-PAGE of a typical WT pyocin sample that was used in solution biophysics experiments. All bands correspond to known pyocin proteins, and no detectable impurities are present. The proteins are identified with their trivial names or locus number in *P. aeruginosa* PAO1 genome (e.g., 618 stands for PA0618). PA0616 (the central spike protein, MW = 19.4 kDa, three copies per particle) and PA0627 (MW = 7.5 kDa) are not visible in this SDS-PAGE. (**B**) The killing activity of the WT pyocin and the four mutants with an insertion of one (1aa) to four (4aa) amino acids in the inter-strand linker is evaluated by a double agar overlay spot assay. In a dilution series up to a certain concentration, active pyocins “burn” a visible spot on a lawn of a sensitive *P. aeruginosa* 13s strain by lysing a large fraction of cells in that spot. A fragment of a Coomassie-stained SDS-PAGE centered on the pyocin sheath band shows the relative amount of pyocins in each sample. “Ctrl” stands for cells that contained an empty vector and did not produce pyocins (negative control sample). (**C** and **D**) EM images (negative staining) of extended and contracted WT pyocin particles, respectively, used in CD, DSC, and ITC measurements.

To gain further insight into the sheath contraction process, we probed the energetics of the system via a series of solution biophysics experiments (Fig. 6). For this, a protocol for the purification of pyocin particles of high purity in the extended and contracted states has been developed (Fig. 7). We found that pyocins contract in a narrow interval of temperatures near ~70°C. Contraction could also be triggered by an acidic buffer (pH <3.0). This made it possible to measure the enthalpy of heat- and pH-triggered contraction with the help of differential scanning calorimetry (DSC) and isothermal titration calorimetry (ITC).

DSC curves of extended and contracted pyocins contained a positive peak in the 60° to 80°C interval. No morphological changes in the contracted specimen were associated with this transition; therefore, this event likely corresponded to the denaturation of a baseplate or a neck component. The contribution of this transition could be accounted for by subtracting the contracted particle DSC curve from that of the extended particle. The resulting enthalpy of sheath contraction was −13.6 ± 1.2 kcal/mol per subunit (i.e., per sheath subunit) (Fig. 6A).

The enthalpy of pH-induced contraction was measured by titrating a pyocin sample having a total ionic strength of ~2 mM into a cell that contained 50 mM glycine-HCl (pH 2.5). The contribution of solvation was accounted for by comparing the enthalpies of the extended and contracted pyocins belonging to the same biological replicate and dialyzed into the same low–ionic strength buffer. In this case, the enthalpy of contraction was −10.1 ± 1.6 kcal/mol per subunit (Fig. 6B). Thus, the experimentally measured enthalpy associated with sheath contraction is nearly equal to the free energy difference calculated by the DMAD procedure (−13.2 kcal/mol per subunit; Fig. 5A), showing that the entropic contribution to contraction is small, further validating the DMAD approach.

**Fig. 8:**
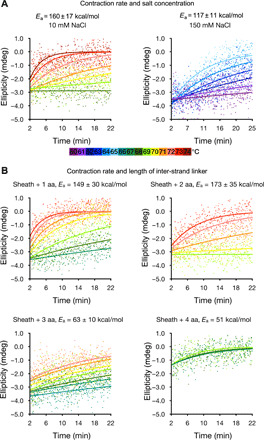
CD spectroscopy of heat-induced contraction. (**A**) Heat-triggered contraction of WT pyocin measured by CD at a wavelength of 203 nm at various temperatures in buffers with two different salt concentrations. The temperature is color-coded according to the color bar given below the panels. The data point series are fitted with exponential functions. Each color corresponds to an average of three technical replicates. (**B**) Heat-triggered contraction of pyocin mutants carrying one, two, three, and four additional amino acids in the inter-strand linker. The color code is the same as in (A).

The folds of the sheath subunit in the extended and contracted states are very similar (Fig. 1B), but their spatial arrangement (the helical symmetry) (Fig. 1, A and C to F), the contacts with each other (Fig. 1, C to F), and the conformation of the inter-strand linker are different (Fig. 1, C to F). The synergetic combination of these effects results in small but detectable differences in the circular dichroism (CD) spectra of contracted and extended pyocins (Fig. 6C). This property made it possible to monitor heat-induced contraction in a solution ensemble in real time. This process was found to be a first-order reaction with a temperature-dependent rate (Fig. 8A). Assuming the Arrhenius model and a temperature-independent activation energy, the logarithm of the turnover rate is inversely proportional to the reaction’s temperature with a coefficient of −*E*_a_/*R*, where *E*_a_ is the activation energy and *R* is the universal gas constant. Such a measured activation energy was found to be 160 ± 17 kcal/mol per particle in a low-salt buffer and decreased to 117 ± 11 kcal/mol per particle as the salt concentration increased (Fig. 6D). This is in qualitative agreement with the DMAD-derived value of 201 ± 12 kcal/mol per particle, which was calculated for the system in pure water (Fig. 5A).

### Inter-strand linker length affects the activation energy

To further validate the results of biophysical experiments and DMAD modeling, we examined the activation energies of pyocin mutants with altered inter-strand linkers. We reasoned that lengthening these linkers, most of which are fully stretched in the transition state (Fig. 5C), should allow the structure to sample a larger parameter space of available contraction pathways, resulting in a lower activation energy (*31*).

A full, quantitative characterization of the contraction of sheath mutants with linker insertions by DMAD requires the knowledge of the structure of these linkers in both end states, which is unavailable. However, considering the geometry of the system (the linkers run at various angles to the long axis of the particle), two- and four-residue insertions into the inter-strand linker will allow for the maximum subunit COM separation to increase by ~4 and ~8 Å, respectively. In the DMAD procedure, this is achieved by decreasing the *k*_2_ constant to 1.4 × 10^−4^ and 8 × 10^−5^ Å^−2^, respectively. The resulting activation energies are progressively lower, albeit within the measure of uncertainty: 192 ± 14 and 186 ± 14 kcal/mol, respectively (Fig. 5A, inset, solid lines).

Experimentally, mutants with insertions of one, three, and four amino acids in the inter-strand linkers were progressively less stable, contracted at lower temperatures (Fig. 6E), and had lower activation energies than the wild-type (WT) structure (Figs. 6F and 8B). The mutant with a two-residue insertion was more stable and had a higher activation energy most likely due to a pleiotropic effect. The four-residue insertion mutant was insufficiently stable to withstand the rigorous purification procedure required for solution biophysics experiments, and its contraction kinetics could only be recorded for three temperature points.

All sheath mutants were functionally active (Fig. 7B). The killing capacities of mutants with one to three residue linker insertions were similar to that of the WT (Fig. 7B), while the four-residue insertion mutant was less active. The value of the activation energy, which determines the stability of the particle, affects the shape of the DMAD-derived free energy profile in the beginning of the contraction trajectory, whereas the force needed to penetrate the membrane, which likely determines the killing capacity, is dictated by the shape of the free energy profile at the end of the contraction process (Fig. 5A and Discussion). Hence, the comparable activities of the sheath linker insertion mutants agree with the DMAD-predicted behavior of the system.

One explanation for the nonlinear behavior of these mutants is that the inter-strand linker insertions affected both the inter- and intra-strand linkers. The DMAD methodology allows us to examine this supposition. By reducing the optimal values of the intra- and inter-strand constants by 5 and 10% (*k*_1_ from 0.7 to 0.665 and 0.63 and *k*_2_ from 2.5 × 10^−4^ to 2.375 × 10^−4^ and 2.25 × 10^−4^ Å^−2^) for the two- and four-residue insertion, respectively, while keeping *D* unchanged results in activation energies of 173 ± 25 and 161 ± 16 kcal/mol (Fig. 5A, inset, dashed lines). Not only do the activation energies decrease but also the transition state intermediate shifts to an earlier, more extended state in the contraction pathway. Such a state is likely to be attained at a temperature lower than that of the WT, as observed experimentally.

### Single-molecule imaging and force measurements of sheath extension

**Fig. 9:**
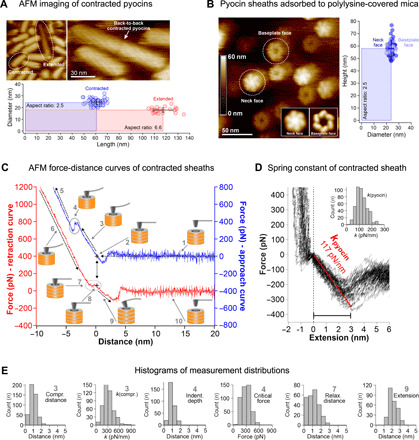
Probing physical properties of the pyocin sheath with AFM. (**A**) AFM images of a pyocin sample adsorbed to the mica surface. The AFM-derived dimensions of the extended and contracted species (bottom) match those recorded in EM. (**B**) AFM images of contracted pyocin sheaths standing vertically on a polylysine-treated mica surface (left subpanel). The baseplate face can be clearly distinguished from the neck face. The inset shows slightly enlarged images of the neck and baseplate faces obtained by averaging multiple images and applying sixfold symmetry. Right: The dimensions of the contracted sheaths in both orientations measured by AFM. (**C**) AFM force-distance curves of contracted sheaths. The cantilever approach and retraction curves are shown in blue and red, respectively. For clarity, they are plotted with an offset along the *y* axis. The following five stages were registered during approach: (1) The tip vibrates freely before contacting the particle; (2) attractive interaction between the tip and the sheath; (3) compression of the sheath; (4) insertion of the tip into the sheath channel; (5) bending of cantilever. Retraction contained the following five stages: (6) straitening of cantilever (inverse of 5); (7) relaxation of the cantilever at zero force; (8) zero-distance adhesive force; (9) extension of the pyocin sheath; (10) the tip separates from the sheath and vibrates freely. All data were calculated by eliminating cantilever deflection. (**D**) Representative pyocin extension curves, with a linear fit of the extension spring constant *k*_pyocin_ of ~117 pN/nm (red line) and a histogram of the spring constant distribution (inset). (**E**) Distributions of various parameters measured by AFM in stages 3, 4, 7, and 9 of the force-distance curve [see (C)] are shown in the form of histograms. All measurements were performed for two biological replicates.

To understand the energetics of the system at the very end of the contraction process and to probe contraction in a single-particle regime, we measured the elastic properties of fully contracted sheaths by atomic force microscopy (AFM). When imaged by high-speed AFM (*32*), the structural features and dimensions of pyocins adsorbed to the mica surface matched those found in high-resolution cryo-EM studies (*5*, *27*), showing that the interaction with mica does not disturb the structure (Fig. 9A). Functionalizing the mica substrate with polylysine made it possible to orient tubeless contracted sheaths vertically (Fig. 9B). These particles displayed two distinct ends: a left-handed windmill hexameric structure with a ~10-nm-wide opening and a closed cap structure, which corresponded to the baseplate and neck faces of the contracted pyocin, respectively (Fig. 9B) (*27*). Following image-based targeting of individual pyocins exposing their open face, the AFM was set to acquire force-distance cycles (Fig. 9C) by inserting the tip into the sheath opening and measuring the spring constant by stretching the particle during tip retraction (Fig. 9D).

A typical force measurement cycle consisted of 10 stages (Fig. 9C). In stage 1, the tip approached the contracted sheath, and no interaction force was detected until attractive (likely van der Waals) forces occurred at ~1-nm tip-sheath separation. A cantilever-sheath contact at zero force followed (stage 2, black cross on blue trace). Upon further approach, the cantilever reported a repulsive force regime (stage 3), which corresponded to the compression of the sheath (*k*_compression_, ~310 pN/nm) (Fig. 9E). The compression distance, which constituted the difference between the displacements of the piezo stage (~3.5 nm) and the cantilever deflection (~2.5 nm), was only ~1 nm or ~1.6% of the ~60 nm height, indicating that the contracted sheath was essentially incompressible. Upon reaching a load of several hundred piconewtons on the cantilever (*F*_critical_, ~290 pN), a relaxation (stage 4) was observed in ~70% of the approach curves (*d*_indent_, ~0.8 nm). We interpreted this event as the tip sliding into the sheath channel. Stage 5 described another linear repulsive force regime (with an up to ~2-nN applied force) with a steeper slope that matched that of the cantilever pushing against the bare mica support as recorded in control measurements before and after the stretching experiments. In stage 6, the cantilever was retracted and straightened. Upon complete cantilever relaxation, a zero-force regime was observed (stage 7) that spanned ~1 nm (*d*_relaxation_, ~1.1 nm) (Fig. 9E). We interpreted this regime as the reverse process of stage 4, since the relaxation distance is very similar to the ingrain distance and terminates precisely at the point of contact (stage 2) in the approach curve (two-headed dashed arrow). Similar to stage 4, this regime was not found in all curves, and/or it varied in length. When the tip-sample separation distance was further increased, beyond the point of the initial tip-sample contact in the approach (stage 8), an adhesive force regime was detected (stage 9). In this regime, the cantilever is stretching the sheath. The sheath could be extended by an average of ~2 nm (*d*_extension_, ~2.1 nm), upon which the adhesive force on the cantilever reached ~250 pN (Fig. 9E). Upon further extension, the nonspecific bond between the cantilever and the sheath broke, and the cantilever snapped back to its relaxed zero-force state (stage 10). Force curve cycles could be repeated on the same particle several times until it fell over or disintegrated (Fig. 9D). Stage 9 describes the force/extension response of the pyocin sheath in its last nanometers of contraction. The spring constant of the contracted pyocin sheath is 117 ± 20 pN/nm (Fig. 9D).

### DMAD-derived force profile of the contraction reaction contains two phases

**Fig. 10:**
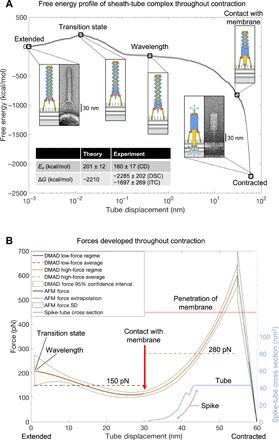
Energies and forces developed by the pyocin sheath-tube complex throughout contraction. (**A**) Evolution of the DMAD-derived free energy of the pyocin sheath-tube complex throughout contraction with key structural intermediates shown in schematic form (see Fig. 1A for color key). The thin lines tracing the free energy curve are the SD of error associated with the extrapolation from the 12-layer fragment (Fig. 4E) to the full-length pyocin structure. The conformations of the end states are visualized by negative stain EM of pyocin particles incubated with the *P. aeruginosa* 13s outer membrane fragments. The inset table compares the energetics of the DMAD methodology with biophysical experiment. The energetic calculations presented in this figure are performed only for the sheath-tube complex. The membrane bilayer, the baseplate, and other components are shown for geometric and illustrative purposes only. (**B**) Evolution of forces developed by the full-length pyocin throughout contraction. The force curve is a negative derivative of a fourth-order polynomial fitted to the free energy profile (A) (Fig. 5A). The error represents a 95% confidence interval for the DMAD-derived forces and is a result of the standard propagation of error from the derivative of a polynomial fit (*33*). The extension force of contracted pyocin sheaths is derived from the spring constant measured by HS-AFM (Fig. 9D). The light blue curve shows the evolution of the cross-sectional area of the spike-tube complex as it crosses the membrane plane (fixed at the initial contact point) during sheath contraction. The cross-sectional area was extracted from atomic coordinates by calculating the polygonal area of horizontal slices of the spike-tube complex (*27*).

The excellent agreement between the predicted energetics of sheath contraction and experiment allows us to speculate about the forces developed by the particle throughout contraction from the DMAD theory (Fig. 10). This force can be estimated by taking the negative derivative of the free energy with respect to tube displacement (Fig. 10, A and B).

The DMAD-derived force profile can be divided into three parts: an initial regime (the first ~1 nm of tube motion), a low-force regime (~1 to 30 nm of tube motion), and a final high-force regime (~30 to 60 nm of tube motion) (Fig. 10B). The scale of tube motion in the first regime is too small for the force to be evaluated by DMAD. The second regime is characterized by a near-constant force of ~150 pN, which is a consequence of the wave-like propagation of contraction. In this regime, the tube moves toward the membrane but does not yet interact with it, and the force is accordingly low. In the final regime, as the surface area of sheath subunits engaged in interfacial interactions increases markedly, the force grows and eventually exceeds 500 pN. At the very end of the contraction process, tube displacements sampled by DMAD are too coarse to accurately evaluate the derivative and therefore the forces generated. AFM measurements show that the sheath behaves like a stiff spring, and the force decreases to zero as the sheath reaches the contracted state. The DMAD-derived and extrapolated AFM force curves intersect at ~600 pN, which likely represents the maximum force developed by the pyocin sheath. Notably, the DMAD-derived force of sheath contraction increases in line with the increase in the cross section of the spike-tube complex as it is driven through the cell membrane (Fig. 10B). The average pressure across the cross section of the tube is ~56 atm.

As DMAD is a deterministic method that finds the most probable path in the conformational space by using a principle of least action type of approach, the main source of errors in its application to pyocin contraction is in the extrapolation from the 12-layer pyocin fragment to the full-length structure. The relationship between a subunit’s contracted fraction and its contribution to the total energy varies slightly depending on the layer. We express this variability via an SD (Fig. 4E). This error is then propagated according to the law of propagation of uncertainties (*33*) to the energetics of the full-length structure where it is very small (Fig. 10A) and to the force profile where it becomes more noticeable (Fig. 10B).

## DISCUSSION

### General applicability of the DMAD approach

Here, we introduced a new coarse-grained modeling methodology (DMAD) that makes it possible to calculate the free energy profile of a physicochemical reaction for a multimillion-atom complex. The DMAD method is innovative in that it can provide physically realistic transition intermediates for systems that are too large for a conventional MD study (*34*). DMAD can also be used to bootstrap MD simulations by providing a set of physically feasible reaction coordinates, the finding of which often constitutes a problem in and of itself (*35*).

DMAD can be applied to any system where conformational transitions are dominated by rigid body motions of individual protein subunits such as the contraction of the sheath or the transformation of the baseplate in CISs (*29*), maturation of virus capsids (*36*), and the rotation of the stator units in the bacterial flagellum (*37*). In these cases, the first step is to determine the range of allowed motions that preserve the quaternary structure of the system. This allowed space of transitions can then be used to define a range of physical parameters such as the “springs” used in the pyocin sheath contraction model (Figs. 1E, 2B, and 3B). The allowed space of transitions can then be systematically sampled as finely or coarsely as desired, resulting in multiple sets of transition intermediates. Next, the energetics of the transition intermediates can be evaluated by a solvent-accessible surface area (SASA)–based approach (such as PISA) or by other methods if the system is sufficiently small (*38*–*40*). Last, the local region of optimal parameters can be further sampled to find the most energetically favorable transition pathway so that the scale of expected values is determined and an appropriate experimental methodology is selected.

One of the main sources of uncertainty in the DMAD approach is the validity of the SASA-based calculations of free energy. While solvation energies are linearly related to the SASA on the macroscale, microscale calculations require a thorough analysis of multibody correlations between the solute and the molecular surface of the protein (*41*, *42*). SASA-based methods work particularly well when the interfaces being analyzed are large (*43*) and rigid (*44*, *45*), as is the case for the pyocin sheath-tube complex. To evaluate the implicit error in approximating solvation energies using SASA methods, we applied PISA to a well-studied system, the association of the proline-rich peptide p41 with the Src homology 3 domain of a tyrosine kinase (Protein Data Bank code: 1BBZ) (*46*). The results showed an association free energy of −8.2 kcal/mol, which is comparable to MD-derived values in the range of −7.7 to −7.8 kcal/mol (*47*) and in agreement with solution biophysics experimental values in the range of −7.7 to −8.0 kcal/mol (*46*, *48*, *49*). Thus, PISA can provide accurate association energy estimates not only for large interfaces as in the pyocin system but also for smaller complexes.

### Additional observations supporting DMAD-derived energy and force profiles

The activation energy of the sheath-tube system is ~160 kcal/mol, which manifests in high stability at room temperature. In vivo, contraction is triggered by the binding of multiple tail fibers to cell surface receptors. The immobilization of the tail fibers reduces their conformational entropy, which likely results in a positive Δ*G* contribution and promotes a conformational change of the baseplate. The latter brings the sheath to the transition state, which then quickly contracts. Accordingly, particles with multiple fibers bound to the cell surface, and extended sheaths are rarely imaged (*30*), likely due to their transient nature and very short lifetime.

This line of arguments is further supported by the DMAD-derived force profile of the contraction reaction and by EM imaging of pyocins (Fig. 10A) and phages T4 and P1 (*30*, *50*). Attachment of tail fibers to the cell surface positions the baseplate ~30 nm away from the lipid membrane (Fig. 10A). Therefore, the tube does not come in contact with the cell membrane until roughly halfway through the contraction process and thus requires little force to traverse the first ~30 nm. Accordingly, in the DMAD-derived force profile of the contraction reaction, the initial force is low (Fig. 10B). However, upon the tube touching the membrane, the force increases as the cross section of the spike-tube complex, which is driven into and across the membrane, increases (Fig. 10B).

This study establishes a relationship between the functional requirements, physical parameters, and the structure of a CIS. Because of the wavelike nature of the propagation of contraction, CISs that are longer than the contraction wavelength have similar activation energies and generate similar forces for membrane puncture by the spike-tube complex. This provides a rationale for the length of the pyocin particle: It is built long enough to develop a force necessary for membrane penetration but no larger, as beyond this point, additional sheath subunits do not lead to greater forces. This result is directly applicable to the function of T6SS and explains how T6SS organelles, which vary greatly in length (both within a single organism and between organisms) and can be as long as 5 μm (~37 times longer than the pyocin) (*51*, *52*), are activated by a baseplate that resembles that of pyocin or T4 phage (*29*, *53*–*55*).

## MATERIALS AND METHODS

### The DMAD procedure

The contraction process was initiated by a small perturbation of baseplate-proximal sheath subunits (which constitutes ~1/1000th of the total motion of the subunit as it travels from the extended to the contracted state). This perturbation then propagated iteratively to all other subunits according to the algorithm described in the “The equations of motion of sheath subunits (propagation of contraction) in pseudocode” section below (data S1). The algorithm was implemented for a total of 100 (*k*_1_, *k*_2_, *D*) parameter sets (Fig. 3B and data S2).

In the laboratory reference frame, each subunit moved along the conical cylindric geodesic connecting the COMs of the subunit in the extended and contracted conformations (Fig. 2A). This motion was modulated by a velocity-like term that corresponded to the fraction of the geodesic that an individual subunit traversed during one iteration. Several analytic expressions of the velocity curve that had vanishing velocities at the two terminal points have been tested. As none of these forms substantially lowered the activation energy or made the overall profile smoother, a parabolic form was used. The maximum velocity comprised 5% of the geodesic path (*v*_max_ = 0.05).

To account for small differences in the fold of the sheath subunit in the extended and contracted states (Fig. 1B), a morphing trajectory containing 99 intermediates of the sheath subunit spanning its extended (conformation 0) and contracted (conformation 100) states was generated with the help of UCSF Chimera (*56*). Accordingly, when the contracted fraction of a subunit was *N*% during a simulation, the coordinates of the *N*th morphing intermediate were used as the starting conformation of the sheath fold.

As any perturbation (no matter how small) to the position of a subunit in the sheath structure resulted in a broken connectivity of the sheath’s mesh, it had to be rebuilt. This task was executed with the help of a semiautomated procedure using Coot (*57*) and other programs from the CCP4 package (*58*). The geometry was improved, and possible clashes were removed with the help of Cartesian dynamics as implemented in the Phenix software package (*59*). The Phenix protocol is a fast, crude version of MD that lacks attractive forces. Dynamic runs had to be kept short and not run at high temperatures. In practice, 400 timesteps of dynamics at 150 K was best for regularizing geometry and resulted in a root mean square deviation of <1 Å for the complex. The resulting structures were of good quality (table S1) (*28*).

Each contraction trajectory contained ~100 intermediate structures. The tube and sheath are linked to each other via a capping protein. Thus, the motion of the tube was derived from the trajectory of the uppermost sheath subunits. The free energy of the solvent-exposed and buried surfaces was calculated with PISA (*26*).

### The equations of motion of sheath subunits (propagation of contraction) in pseudocode

Let *k*_1_, *k*_2_, *D* be the intra-strand coupling constant, the inter-strand coupling constant, and the drag parameter, respectively.

Let $r_{\text{ext}}^{i},r_{\text{cnt}}^{i}$ be the radius of the COM of the *i*th subunit (*i* ∈ {1,2, …,12}) in the extended and contracted states, respectively.

Let θ*^i^* be the angle between the COMs of the *i*th subunit in the extended and contracted states in the *x*-*y* plane (such that the bottom sheath layer COM is centered on the origin and subsequent layer COMs lie on the +*z* axis).

Let $\theta_{0}^{i}$ be the angle that describes the location of the COM of the *i*th subunit in the extended state in the *x*-*y* plane.

Let $z_{\text{ext}}^{i},z_{\text{cnt}}^{i}$ be the height of the COM (projection onto the *z* axis) of the *i*th subunit in the extended and contracted states, respectively.

Let $b_{j}^{i}$ be the scalar distance between the COMs of the *i*th subunit and the subunit connected to it by the inter-strand linker at the *j*th iteration of the algorithm, such that $b_{0}^{i}$ is the distance between COMs in the extended state.

Let $s_{j}^{i}$ be the scalar difference between $b_{j}^{i}$ and $b_{0}^{i}$, such that

$$s_{j}^{i}=\{\begin{array}{c} b_{j}^{i}-b_{0}^{i},b_{j}^{i}>b_{0}^{i}\\ 0,b_{j}^{i}\leq b_{0}^{i} \end{array}$$

Let $v_{j}^{i}$ be the scalar “subunit velocity” of the *i*th subunit at the *j*th iteration of the algorithm where the maximum velocity is *v*_max_.

Let $d_{j}^{i}$ be the scalar displacement of the COM of the *i*th subunit between iterations *j* and *j* − 1, such that $d_{1}^{i}=0$.

Let $\lambda_{j}^{i}\in [0,1]$ be the contracted fraction of the *i*th subunit at the *j*th iteration, where λ = 0 represents the extended state, and λ = 1 represents the contracted state.

Let (ω*^i^*, φ*^i^*, κ*^i^*) be the set of polar angles that describe the rotation of *i*th subunit about its COM from the extended to the contracted state. (ω*^i^*, φ*^i^*) describe the axis of rotation in the reference frame of the COM and thus are fixed throughout the trajectory. Therefore, the orientation of the *i*th subunit at the *j*th iteration of the algorithm can be described by $\left(\omega^{i},\varphi^{i},\kappa_{j}^{i}\right)$, where $\kappa_{j=0}^{i}=0$ in the extended state.

Let $r_{j}^{i},z_{j}^{i},\vartheta_{j}^{i}$ be the radius, height, and azimuth of the COM of the *i*th subunit at the *j*th iteration of the algorithm, respectively.

Let *P* be the initial perturbation factor.

As the algorithm commences, the extended structure is perturbed slightly such that

$$\lambda_{1}^{i}=P{\left(k_{1}\right)}^{i-1}$$

$$\left(\omega^{i},\varphi^{i},\kappa_{1}^{i})=(\omega^{i},\varphi^{i},\lambda_{1}^{i}\kappa^{i}\right)$$

$$r_{1}^{i}=r_{\text{ext}}^{i}+\lambda_{1}^{i}(r_{\text{cnt}}^{i}-r_{\text{ext}}^{i})$$

$$z_{1}^{i}=z_{\text{ext}}^{i}+\lambda_{1}^{i}(z_{\text{cnt}}^{i}-z_{\text{ext}}^{i})$$

$$\vartheta_{1}^{i}=\theta_{0}^{i}+\lambda_{1}^{i}\theta^{i}$$

This defines the initial perturbed state. Now, contraction progresses as follows until the fully contracted state is reached

Set j=2

While $r_{j}^{12}<r_{\text{cnt}}^{12}$:

For ∈{1,2, …,12}:

$$v_{j}^{i}=v_{\text{max}}(-{\left(\lambda_{j-1}^{i}\right)}^{2}+\lambda_{j-1}^{i})$$

end_For

$$\lambda_{j}^{1}=\text{min}(1,\lambda_{j-1}^{1}+\frac{v_{j}^{1}+k_{1}v_{j}^{2}}{1+k_{1}}-k_{2}{\left(s_{j-1}^{2}\right)}^{2}-Dd_{j-1}^{1})$$

For ∈{2, …,11}:

$$\lambda_{j}^{i}=\text{min}(1,\lambda_{j-1}^{i}+\frac{v_{j}^{i}+k_{1}v_{j}^{i-1}+k_{1}v_{j}^{i+1}}{1+2k_{1}}+k_{2}({\left(s_{j-1}^{i}\right)}^{2}-{\left(s_{j-1}^{i+1}\right)}^{2})-Dd_{j-1}^{i})$$

end_For

$$\lambda_{j}^{12}=\text{min}(1,\lambda_{j-1}^{12}+\frac{v_{j}^{12}+k_{1}v_{j}^{11}}{1+k_{1}}+k_{2}{\left(s_{j-1}^{12}\right)}^{2}-Dd_{j-1}^{12})$$

For ∈{1,2, …,12}:

$$\left(\omega^{i},\varphi^{i},\kappa_{j}^{i})=(\omega^{i},\varphi^{i},\lambda_{j}^{i}\kappa^{i}\;\right)$$

$$r_{j}^{i}=r_{\text{ext}}^{i}+\lambda_{j}^{i}(r_{\text{cnt}}^{i}-r_{\text{ext}}^{i})$$

$$z_{j}^{i}=z_{\text{ext}}^{i}+\lambda_{j}^{i}(z_{\text{cnt}}^{i}-z_{\text{ext}}^{i})$$

$$\vartheta_{j}^{i}=\theta_{0}^{i}+\lambda_{j}^{i}\theta^{i}$$

bji=(rji cos(ϑji)−rji−1 cos(ϑji−1+π3))2+(rji sin(ϑji)−rji−1 sin(ϑji−1+π3))2+(zji−zji−1)2

$$d_{j}^{i}=\sqrt{{\left(r_{j}^{i}\;\text{cos}(\vartheta_{j}^{i})-r_{j-1}^{i}\;\text{cos}(\vartheta_{j-1}^{i})\right)}^{2}+{\left(r_{j}^{i}\;\text{sin}(\vartheta_{j}^{i})-r_{j-1}^{i}\;\text{sin}(\vartheta_{j-1}^{i})\right)}^{2}+{\left(z_{j}^{i}-z_{j-1}^{i}\right)}^{2}}$$

end_For

j=j+1

end_While

### Extrapolation of the 12-layer fragment contraction pathway to the full length sheath

The method of extrapolating results of the 12-layer simulations to generate full-length models of sheath contraction consists of two steps:

1) Use the optimal (*k*_1_, *k*_2_, *D*) parameters found in the global search for the 12-layer fragment to calculate the position of every subunit in the full-length structure for each contraction intermediate.

2) Analyze the distance between sheath subunit COMs in these intermediates and reject pathways with values greater than 55 Å (separations of greater than 55 Å resulted in the disintegration of the handshake domain, thus breaking the native sheath mesh; Fig. 4D).

In addition, for step 1, the following properties of the system had to be taken into account.

The terms $k_{2}({\left(s_{j-1}^{i}\right)}^{2}-{\left(s_{j-1}^{i+1}\right)}^{2})$ and $Dd_{j-1}^{i}$ in the contraction fraction parameter formula

$$\lambda_{j}^{i}=\text{min}(1,\lambda_{j-1}^{i}+\frac{v_{j}^{i}+k_{1}v_{j}^{i-1}+k_{1}v_{j}^{i+1}}{1+2k_{1}}+k_{2}({\left(s_{j-1}^{i}\right)}^{2}-{\left(s_{j-1}^{i+1}\right)}^{2})-Dd_{j-1}^{i})$$

vary with the length of the sheath due to their dependence on the path of the subunit along its geodesic trajectory. For this reason, the extrapolation from the 12-layer fragment to the full-length sheath required rescaling of the *k*_2_ and *D* parameters as follows (*k*_1_ was not rescaled as *v* does not depend on the path):

$$k_{2}\;\text{was rescaled by a factor of}\;X^{2},$$

D was rescaled by a factor of X,

where *X* = 1/2.44 is the ratio of the average distance traveled by all subunits on their conical geodesic trajectories in the 12-layer simulation relative to the distance traveled in the full-length simulation.

### Purification of pyocins for biophysical experiments

Pyocin particles were produced in *Escherichia coli* using a pETcoco-1–based plasmid that contained the entire cluster of R2 pyocin genes (*Pseudomonas aeruginosa* PAO1 genes *pa0610* to *pa632*) including the regulatory genes *prtN* (*pa0610*) and *prtR* (*pa611*) and the lysis cassette (*pa0629* to *pa0632*). The plasmid was created by Scholl and colleagues (*60*) (plasmid pSW192, AvidBiotics Corp.), and its design and construction are described elsewhere.

In a medium free from arabinose, the cells carry one or two copies of the pSW192 plasmid, and the cluster is completely inhibited. The addition of arabinose increases the copy number of the pSW192 plasmid, and this activates the entire operon, including lysis genes. Thus, production of pyocin particles can be triggered by the addition of arabinose. Newly assembled pyocin particles are released from the cells by lysis. These particles were morphologically indistinguishable from pyocins produced by PAO1 treated by mitomycin C. The killing activity was quantified by spot killing assay on a sensitive *P. aeruginosa* 13s strain. EM showed that the “recombinant” *E. coli*–produced pyocins were all extended, unlike those purified from *Pseudomonas* that contain a fraction of contracted particles, possibly because *E. coli*–produced pyocins are never exposed to *Pseudomonas* cell fragments during the purification procedure.

Eight liters of *E. coli* BL21ΔAra (*60*) freshly transformed with pSW192 was grown in Lennox LB medium (Thermo Fisher Scientific, catalog no. BP1427-500) supplemented with chloramphenicol at 11 μg/ml (Thermo Fisher Scientific, catalog no. BP904-100) in eight 4-liter Erlenmeyer flasks at 37°C and 240 rpm to an optical density (OD) of 1.0 at 600 nm. Five milliliters of 20% arabinose (Thermo Fisher Scientific, catalog no. AAA1192118) and 5 ml of 80% glycerol (Thermo Fisher Scientific, catalog no. G33-500) were added to each flask, the temperature was decreased to 30°C, and the cells were incubated at this temperature overnight. Debris and residual bacteria were removed from the lysate by centrifugation at 15,000*g* for 30 min in a F9-6x-1000 rotor (Thermo Fisher Scientific, catalog no. 09-606-1075). The supernatant was then filtered through a 1V paper filter (GE Healthcare, catalog no. 1201270), and 4 mg of deoxyribonuclease I (DNase I) and 4 mg of ribonuclease A (RNase A) (MilliporeSigma, catalog nos. 69182 and 556746, respectively) were added to it. The clarified lysate was supplemented with 240 g of NaCl (dry powder) and 800 g of PEG 8000 (dry flakes) (Thermo Fisher Scientific, catalog nos. 18-606-422 and BP233-1, respectively). The chemicals were dissolved by stirring, and then the mixture was incubated at 4°C overnight to ensure complete precipitation of pyocins. Pyocins were pelleted at 15,000*g* for 15 min in a F9-6x1000 rotor. The pellets were resuspended in 80 ml of SM buffer [8 mM MgCl_2_, 100 mM NaCl, and 50 mM tris-HCl (pH 7.5)] with DNase I and RNase A at 1 μg/ml each. The cell membrane and other associated impurities were removed by the addition of 80 ml of chloroform (Thermo Fisher Scientific, catalog no. C298-500) and centrifugation in 50-ml Falcon tubes at 15,000*g* for 15 min. The pyocin-containing aqueous phase was collected, and pyocins were pelleted at 100,000*g* for 2 hours in a T29-8x50 rotor (Thermo Fisher Scientific, catalog no. 75-003-009). The pellets were dissolved in 4 ml of SM buffer on an orbital shaker at 100 rpm overnight at 4°C. Undissolved material was removed by centrifugation at 15,000*g* for 5 min in a microcentrifuge at room temperature. The soluble fraction was divided into halves, and each part was then loaded onto a premade step gradient of 10%, 20%, 30%, 40% (2 ml each), and 60% sucrose (3-ml bottom cushion) in SM buffer. The tubes were then centrifuged at 100,000*g* for 1 hour in a SW40Ti rotor (Beckman Coulter). Bands containing pyocin particles were located in the upper part of the gradient and were visible by eye. These bands were extracted from both tubes, combined, and dialyzed against two changes of 0.1× SM buffer. The concentration of the dialyzed sample was determined on the basis of the adsorption at 280 nm and brought to 5 mg/ml. To obtain a sample of fully contracted pyocins, 15 μl of 3 M glycine-HCl (pH 2.5) was added to 3 ml of purified extended pyocin sample at 5 mg/ml, and glycine was dialyzed out using two changes of the same 0.1× SM buffer.

### Design of sheath mutants

Additional residues were introduced into the inter-strand linker of the PA0622 sheath protein between Gly^23^ and Ser^24^ with the help of a modified allelic exchange procedure (*61*). For each mutant, two overlapping ~1-kb-long fragments carrying the mutation were amplified by appropriate pairs of primers (table S2) and pSW192 as the template. The flanking primers AE21M1F and AE21M2R were common for all four linker mutants. The exchange donor vectors were assembled by NEBuilder reaction (New England Biolabs, Ipswich, MA) on the backbone of the pWM91 plasmid (*62*) in which the ampicillin resistance gene was replaced with the kanamycin resistance gene. The recipient plasmid pSW192 was maintained in RecA+ *E. coli* 4s strain (*63*). The donor vectors were transformed into the MFDpyr *E. coli* strain (*64*), and conjugation between the donor and the acceptor strains was performed on LB agar plates overnight at 37°C. Selection for recombination products was done on LB agar plates supplemented with kanamycin at 50 μg/ml. Counterselection for excision products, pSW192 and the mutation carrying plasmids, was done on agar plates with 1% tryptone, 0.5% yeast extract, and 5% sucrose (MilliporeSigma, Burlington, MA) overnight at room temperature. Colony screening was done by polymerase chain reaction. The presence of mutations was confirmed by Sanger sequencing.

### Killing assay for pyocin sheath mutants

The WT and mutant pyocins were expressed in the *E. coli* strain BL21Δ*ara*Δ*fhuA*Δ*ompF* using the pSW192 plasmid and its sheath mutant derivatives as described above. The cells were allowed to lyse, and the particles were purified as follows. Cell debris were removed by centrifugation at 15,000*g* for 15 min using the F9-6x-1000 rotor (Thermo Fisher Scientific, catalog no. 09-606-1075). The pyocins were pelleted by centrifugation at 100,000*g* for 6 hours using the SW-28 rotor (Beckman Coulter, catalog no. 342204). The pellets were dissolved in SM buffer overnight, and both centrifugation steps (medium and high speed) were repeated. The samples were kept at 4°C throughout the purification. The samples were normalized for their protein concentration according to their ultraviolet absorbance at 280 nm. The killing activity of pyocins contained in the samples was evaluated by a double agar overlay spot assay on a lawn of *P. aeruginosa* 13s cells (R2 pyocin–sensitive strain) (*65*). Double agar overlay plates with bacterial lawns were prepared using a standard procedure (*66*). Cells transformed with an empty pETcoco-1 vector (Sigma-Aldrich, catalog no. 71129) served as a negative control. Since these cells predictably did not lyse after induction, they were disrupted by ultrasound sonication and subjected to the same purification steps as the lysates containing pyocins. The sample content was verified with SDS–polyacrylamide gel electrophoresis (PAGE).

### Preparation of *P. aeruginosa* 13s outer membrane fraction

The outer membrane fraction of *P. aeruginosa 13s* was purified by differential solubilization (*67*) with the help of *N*-lauroylsarcosine sodium salt (Sigma-Aldrich). The cell culture was grown in 50 ml of LB media up to late log phase (OD_600nm_ = 1.0) at 37°C and vigorous shaking. The cells were pelleted by centrifugation at 5000*g* for 5 min. The cells were resuspended in 10 ml of 10 mM tris-HCl (pH 8.0) supplemented with DNase I and RNase A at 1 μg/ml each and disrupted by sonication. The total membrane fraction was pelleted by centrifugation at 100,000*g* for 30 min, resuspended in the same buffer, and pelleted again. The pellet was resuspended in 1% *N*-lauroylsarcosine in 10 mM tris-HCl (pH 8.0) and incubated at 37°C for 30 min with moderate shaking to selectively dissolve the inner membrane. The sample was centrifugated at 100,000*g* for 30 min, resuspended in 10 mM tris-HCl (pH 8.0) and pelleted again, and then lyophilized and weighed, taking into account the presence of the tris buffer in the sample.

### Electron microscopy

Twenty microliters of *P. aeruginosa* 13S outer membrane fragments (10 mg/ml) (in 0.1× SM buffer) was incubated with 20 μl of purified extended pyocin particles (1 mg/ml) (in 0.1× SM buffer) for 20 min. After incubation, 5-μl samples were aliquoted onto plasma-cleaned Electron Microscopy Sciences CF200-CU grids, excess sample was blotted, then were stained twice with 10 μl of 7% uranyl acetate in 50% ethanol solution. The grids were mounted into a JEOL 2100 microscope and imaged at a magnification of ×40,000.

### DSC and ITC

The enthalpy of heat-induced pyocin contraction was measured with MicroCal PEAQ-DSC (Malvern Instruments) and NanoDSC (TA Instruments) microcalorimeters. The sample concentration was 4.5 to 5 mg/ml in 0.1× SM buffer [0.8 mM MgCl_2_, 10 mM NaCl, and 5 mM tris-HCl (pH 7.5)]. The scanned temperature range was from 20° to 130°C. The heating rates for the MicroCal PEAQ-DSC and NanoDSC instruments were 200°C/hour and 120°C/hour, respectively. Calculations of the molar concentration of sheath subunits took into account that the sheath constituted 55.16% of the total mass of the pyocin particle and contains 168 subunits. For instance, in a pyocin sample with a concentration of 5 mg/ml, the molar concentration of sheath subunits is 77 μM (data S2).

A MicroCal PEAQ-ITC microcalorimeter (Malvern Instruments) was used to determine the enthalpy of pyocin contraction induced by exposure to low-pH buffer. The ITC experiment was conducted in an unconventional mode where the pyocin sample was titrated into the cell containing low-pH buffer. The pyocin samples (both the extended and contracted particles) were dialyzed into 0.05× SM buffer [0.08 mM MgCl_2_, 1 mM NaCl, and 0.5 mM tris-HCl (pH 7.5)]. The pyocin concentration in the injection syringe was 5 mg/ml. The cuvette of the calorimeter contained 50 mM glycine-HCl (pH 2.5). The experiments consisted of seven injections, with 60 s in between. The volume of each injection was 5 μl (35 μl of a sample per run in total). The temperature was held constant at 20°C (data S2).

### CD and contraction kinetic measurements

CD spectra and time-course measurements were recorded using a JASCO J-815 CD spectrometer equipped with a Peltier cell. The concentration used for both extended and contracted samples was 0.1 mg/ml in 10 mM NaCl and 10 mM phosphate buffer (pH 7.0). Before all measurements, the condition and conformation of the specimens (extended or contracted) were confirmed by negative stain EM and killing assay on sensitive *P. aeruginosa 13s*. Exposure of an extended pyocin sample to heat for a prolonged time changed its spectrum to a contracted-like one. EM showed that, in such samples, all sheaths were contracted, and some material was aggregated (some baseplates and tubes were stuck together). Exposure of a contracted pyocin specimen to heat did not change its spectrum beyond the noise level, and the sample displayed similar aggregation.

A wavelength of 203 nm was chosen for time-course measurements to maximize the difference between extended and contracted specimen spectra (Fig. 6C). The WT pyocin and its mutants displayed measurable contraction kinetics in a 60° to 74°C range, depending on the buffer composition (data S2).

All measurements were performed with at least three technical replicates of at least two biological replicates (three replicates were used for the WT). Each time-course measurement (every mutant and every de novo purification) required a contracted specimen as a control. These contracted samples were measured for at least two different temperatures and in multiple technical replicates. The contracted specimen curves were averaged and subtracted from the averaged extended specimen curves to obtain the contraction time course for each of the reactions. In all datasets, the first 2 min were trimmed because they corresponded to sample heating, mixing, and equilibration. Data analysis was performed in MATLAB CFTool (MathWorks).

### High-speed AFM

Pyocins were first characterized from side views when physisorbed on mica (Fig. 9A and data S2). The head-on pyocin sheaths (Fig. 9B) were prepared by modifying the mica surface with 0.01% polylysine for 3 min and then depositing the contracted sheaths (from 10 times diluted sample) for 10 min. All images in this study were acquired using a HS-AFM (*32*) (SS-NEX, RIBM, Japan) operated in amplitude modulation mode using optimized scan and feedback parameters at room temperature. Short (8-μm) cantilevers (NanoWorld, Switzerland) with nominal spring constant *k*_c_= 0.15 N/m, resonance frequency of ~0.6 MHz, and a quality factor *Q*_c_∼1.5 in buffer [50 mM tris-HCl (pH 7.5), 100 mM NaCl, and 8 mM MgSO_4_] were used. The energy delivered by a tip-sample interaction can be estimated by $\text{∆}E=(1-\alpha )*k_{c}(A_{o}^{2}-A_{s}^{2})/(2\;Q_{c})$, with α = 0.5 being the ratio of the amplitude reduction caused by the cantilever resonance frequency shift over the total amplitude reduction. To avoid fall over or displacement of the head-on physisorbed pyocin sheaths during AFM imaging, the tip-sample interaction was minimized by using a free amplitude *A*_o_ = 1 nm and a set-point amplitude *A*_s_ ≥ 0.9, respectively. Under such conditions, the energy delivered is ~1.2 *k*_B_*T*, while most of the input energy will be dissipated into the fluid between taps. Both ends of the contracted pyocin sheath, the open baseplate and closed neck end, could be easily identified during imaging. Force spectroscopy measurements were performed following the targeting of the baseplate end of single pyocin sheaths only in HS-AFM imaging mode. Upon centering on the opening in a single pyocin sheath, the setup was switched into force measurement mode, and approach-retract cycles were acquired at 150 nm/s velocity.

### Molecular graphics

Figures 1 (B to F) and 4C and movies S1 and S2 were created using UCSF Chimera (*56*). Figures 4B and 5C were created with the help of UCSF ChimeraX (*68*).

## SUPPLEMENTARY MATERIALS

Supplementary material for this article is available at http://advances.sciencemag.org/cgi/content/full/7/24/eabf9601/DC1

## Appendix

View/request a protocol for this paper from *Bio-protocol*.
